# Defective microglial development in the hippocampus of *Cx3cr1* deficient mice

**DOI:** 10.3389/fncel.2015.00111

**Published:** 2015-03-31

**Authors:** Francesca Pagani, Rosa C. Paolicelli, Emanuele Murana, Barbara Cortese, Silvia Di Angelantonio, Emanuele Zurolo, Eva Guiducci, Tiago A. Ferreira, Stefano Garofalo, Myriam Catalano, Giuseppina D’Alessandro, Alessandra Porzia, Giovanna Peruzzi, Fabrizio Mainiero, Cristina Limatola, Cornelius T. Gross, Davide Ragozzino

**Affiliations:** ^1^Center for Life Nanoscience – Istituto Italiano di Tecnologia@Sapienza, RomeItaly; ^2^Division of Psychiatry Research, University of Zürich, ZürichSwitzerland; ^3^Mouse Biology Unit, European Molecular Biology Laboratory, MonterotondoItaly; ^4^Department of Physiology and Pharmacology, Istituto Pasteur-Fondazione Cenci Bolognetti, Sapienza University of Rome, RomeItaly; ^5^Consiglio Nazionale delle Ricerche – Institute of Inorganic Methodologies and Plasmas, Department of Physics, Sapienza University of Rome, RomeItaly; ^6^Department of Neuropathology, Academic Medical Center, University of AmsterdamAmsterdam, Netherlands; ^7^Istituto di Ricovero e Cura a Carattere Scientifico NeuromedPozzilli, Italy; ^8^Department of Molecular Medicine, Istituto Pasteur-Fondazione Cenci Bolognetti, Sapienza University of RomeRome, Italy; ^9^Department of Experimental Medicine, Sapienza University of RomeRome, Italy

**Keywords:** microglia, CX3CR1, fractalkine, development, rearrangement, potassium currents

## Abstract

Microglial cells participate in brain development and influence neuronal loss and synaptic maturation. Fractalkine is an important neuronal chemokine whose expression increases during development and that can influence microglia function via the fractalkine receptor, CX3CR1. Mice lacking *Cx3cr1* show a variety of neuronal defects thought to be the result of deficient microglia function. Activation of CX3CR1 is important for the proper migration of microglia to sites of injury and into the brain during development. However, little is known about how fractalkine modulates microglial properties during development. Here we examined microglial morphology, response to ATP, and K^+^ current properties in acute brain slices from *Cx3cr1* knockout mice across postnatal hippocampal development. We found that fractalkine signaling is necessary for the development of several morphological and physiological features of microglia. Specifically, we found that the occurrence of an outward rectifying K^+^ current, typical of activated microglia, that peaked during the second and third postnatal week, was reduced in *Cx3cr1* knockout mice. Fractalkine signaling also influenced microglial morphology and ability to extend processes in response to ATP following its focal application to the slice. Our results reveal the developmental profile of several morphological and physiological properties of microglia and demonstrate that these processes are modulated by fractalkine signaling.

## Introduction

Microglia, traditionally known as resident immune cells in the central nervous system (CNS), play essential roles in brain circuit maturation during development, participating in the precise refinement of synaptic connections (Tremblay et al., 2010; Paolicelli et al., 2011; Schafer et al., 2012; Zhan et al., 2014). Microglial cells derive from myeloid lineage and colonize the immature brain during development, progressively acquiring a ramified morphology, commonly associated to surveying activity (Prinz and Mildner, 2011; Walker et al., 2014). Ramified microglia lie very close to each other and have highly motile filopodia-like protrusions of variable shape, defining rarely overlapping niches of competency (Raivich, 2005). Microglial processes continuously sample the extracellular space and contact pre- and post-synaptic elements (Davalos et al., 2005; Nimmerjahn et al., 2005; Wake et al., 2009; Tremblay et al., 2010). Among the functional consequences of this active and continuous surveillance of the developing brain appear to be the shaping neuronal circuits by phagocytosis (Paolicelli et al., 2011) and favoring synaptic maturation (Hoshiko et al., 2012). In damaged brain, microglial processes rapidly rearrange toward the site of injury (Nimmerjahn et al., 2005; Lee et al., 2008). Among soluble factors activating microglial cells, ATP regulates microglial processes extention and ramification in both physiological and pathological conditions (Davalos et al., 2005; Inoue et al., 2007). To reach the site of activating stimuli, microglia retract their cellular processes and rearrange them in a directional manner toward the lesion. In addition, a marked change in ionic channel expression has been observed (Boucsein et al., 2000). Indeed, in acute brain slices non-activated microglia frequently display a small linear conductance, while following nerve lesion (Boucsein et al., 2000) or status epilepticus (Avignone et al., 2008) they transiently express inward and outward rectifier potassium (K^+^) currents (Menteyne et al., 2009; Kettenmann et al., 2011). Ion channels have been involved in many microglial functional properties (Eder, 2005), such as cell volume regulation (Schlichter et al., 1996; Eder et al., 1998), proliferation (Kotecha and Schlichter, 1999), migration (Rappert et al., 2002; Schilling et al., 2004) and cell process extension and retraction (Eder et al., 1998). However, microglia activation is no longer considered an all or none event, but rather a sequence of progressive stages (Ponomarev et al., 2007; Olah et al., 2012; Crain et al., 2013) depending on the balance between pro-inflammatory and anti-inflammatory signals in the surrounding environment (Biber et al., 2007; Lively and Schlichter, 2013).

Chemokines are key molecules in neuron-microglia communication in the developing and mature brain under both physiological and pathological conditions (de Jong et al., 2005). Fractalkine is an important neuron-microglia chemokine signal. In the CNS, fractalkine is expressed by neurons, especially in forebrain structures (Tarozzo et al., 2003), while its receptor, CX3CR1, is expressed uniquely by microglia (Harrison et al., 1998; Jung et al., 2000; Cardona et al., 2006; Mizutani et al., 2012). Recent studies showed that fractalkine-mediated microglia-neuron signaling modulates numerous physiological processes across the lifespan, including the maturation of synaptic connections (Paolicelli et al., 2011; Zhan et al., 2014), neuronal survival (Limatola et al., 2005; Lauro et al., 2008), and synaptic transmission and plasticity (Bertollini et al., 2006; Ragozzino et al., 2006; Piccinin et al., 2010; Maggi et al., 2011; Hoshiko et al., 2012). Mice lacking fractalkine signaling display deficits in hippocampal-dependent learning and memory (Rogers et al., 2011; Zhan et al., 2014) and in microglial properties in barrel cortex (Arnoux et al., 2013). Moreover, reduced microglia colonization during development has been described in hippocampus (Paolicelli et al., 2011) and somatosensory cortex (Hoshiko et al., 2012) in these mice.

In order to better understand the role of fractalkine signaling in microglia physiology and its possible impact on brain development, we studied several functional properties of microglia in acute brain slices from *Cx3cr1* knockout mice across the first postnatal weeks (PNWs). We report that the functional properties of microglia undergo dynamic changes during development and that these changes are absent or delayed in mice lacking fractalkine signaling. These data reveal the highly dynamic nature of microglial maturation during brain development and further highlight the importance of fractalkine signaling in this process.

## Materials and Methods

### Animals and Ethical Approval

For acute slice preparation, *Cx3cr1*^GFP/+^ and *Cx3cr1*^GFP/GFP^ mice were used (Jung et al., 2000). For microglia morphometric analysis in perfused brains, *Cx3cr1*^GFP/+^ and *Cx3cr1*^GFP/KO^ littermates were produced by intercrossing *Cx3cr1*^GFP/GFP^ with *Cx3cr1*^KO/+^ breeders (Haskell et al., 2001). This breeding strategy was required for the progeny to have a single GFP allele, regardless the Cx3cr1 genotype. Such approach was necessary for imaging microglia with similar fluorescent intensity thresholds. Procedures using laboratory animals were in accordance with the international guidelines on the ethical use of animals from the European Communities Council Directive of November 24, 1986 (86/609/EEC). All efforts were made to minimize animal suffering and to reduce the number of animals used, in accordance with the European Communities Council Directive of September 20, 2010 (2010/63/UE).

### Slice Preparation

Acute hippocampal slices were prepared from *Cx3cr1^+/GFP^* and *Cx3cr1^GFP/GFP^* mice (Jung et al., 2000) in the first six PNWs. Animals were decapitated under halothane anesthesia, and whole brains were rapidly immersed for 10 min in chilled artificial cerebrospinal fluid (ACSF) containing (in mM): NaCl 125, KCl 2.3, CaCl_2_ 2, MgCl_2_ 1, NaHPO_4_ 1, NaHCO_3_ 26, and glucose 10 (Sigma Aldrich). The ACSF was continuously oxygenated with 95% O_2_, 5% CO_2_ to maintain physiological pH. Transverse 250 μm hippocampal slices were cut at 4°C with a vibratome (DSK, Kyoto, Japan), placed in a chamber containing oxygenated ACSF and allowed to recover for at least 1 h at room temperature. All recordings were performed at room temperature on slices submerged in ACSF and perfused (1 ml/min) with the same solution in the recording chamber under the microscope. For microglia morphometric analysis from perfused brains, on post-natal day 8 (P8), pups were anesthetized intraperitoneally with Avertin (Sigma-Aldrich, St Louis, MO, USA) and perfused transcardially with 4% paraformaldehyde. Brains were postfixed overnight at 4°C, and sliced afterward on a vibratome (100 μm thick sections; Leica Microsystems, Wetzlar, Germany). Upon DAPI staining, slices were mounted on glass slides and kept for images acquisition.

### Time-Lapse Imaging in Acute Hippocampal Slices

Time-lapse fluorescence determinations were acquired at room temperature (24–25°C) using a customized digital imaging microscope. Excitation of GFP was achieved using a 1-nm-bandwidth polychromatic light selector (Till Polychrome V), equipped with a 150 W xenon lamp (Till Photonics, Germany). Fluorescence was visualized using an upright microscope (Axioskope) equipped with a 40x water-immersion objective (Achroplan CarlZeiss, USA) and a digital 12 bit CCD camera system (SensiCam, PCO AG, Germany). All the peripheral hardware control, image acquisition and image processing were achieved using customized software Till Vision v. 4.0 (Till Photonics. A glass pipette containing adenosine 5′-triphosphate magnesium salt (ATP, 3 mM; Sigma Aldrich) was placed in the stratum radiatum in the center of the recording field. Mg-ATP was pressure applied to the slices (100 ms; 5 psi) with a Picospritzer III (Parker Instrumentation). Changes in GFP fluorescence distribution were monitored by acquiring a fluorescent image every 10 s for 50 min. To quantify the speed of microglial processes rearrangement toward the pipette tip, we measured the increase of GFP fluorescence in a circular area centered on the pipette tip (10 μm radius). At each time point the fluorescence increase in the area was calculated as ΔF = F-F_0_, and then divided for F_0_ (ΔF/F_0_, where F_0_ is the average fluorescence before ATP puff), to normalize the difference in basal GFP fluorescence in slices from the two genotypes. Slices were used from 2 to 7 h after cutting.

### Tracking Analysis of Single Microglial Process

All images were processed using ImageJ software (Schindelin et al., 2012; Schneider et al., 2012). Images stacks were exported as .avi files to enable manual cell processes tracking on the ImageJ “Manual Tracking” plug-in (http://imagej.nih.gov/ij/plugins/track/track.html). To obtain quantitative distributions of tracks parameters, data were analyzed with ImageJ and Origin 7 (OriginLab Co.) software. Stacks were first background subtracted to optimize contrast. To obtain x–y coordinates of single processes, track positions were transferred into a new coordinate system, in which the ATP-containing pipette tip was set as origin (x = 0, y = 0). For each moving process (i), with position vector Ri(t), the change in position from one frame to the next ***[ΔRi(t)]***, and the instantaneous velocity ***[vi(t)]*** were given by ΔRi(t) = Ri(t+Δt) – Ri(t), and vi(t) = ΔRi(t)/Δt respectively, where Δt is the elapsed time among the two frames. The mean velocity of each process was calculated as <v> = dx/dt, expressed in μm/min, defining dx as the mean accumulated distance of each process i sampled within the time interval dt. The position of each process was converted in polar coordinates from Cartesian coordinates. The radial distance r in every pair of successive images was calculated by measuring the Euclidean distance of individual tracks considering r(t)^2^ = (x(t))^2^ + (y(t))^2^. The mean velocity vector was then expressed as in polar coordinates oriented to the center, corresponding to the ATP-containing pipette tip. Displacement is defined as the vector length of the final coordinates from the origin. Directionality is an index of the straightness of the processes trajectory. Index values close to *one* indicates a straight migration trajectory toward the ATP-containing pipette; low values refer to process following a meandering path. Migration directionality was calculated as the ratio of the vector displacement of each process to their accumulated distance (process track) with the help of ImageJ software as previously described (Wu et al., 2012).

### Whole Cell Patch Clamp Recordings

Visually identified GFP-expressing microglial cells were patched in whole-cell configuration in the CA1 stratum radiatum. Micropipettes (4–5 MΩ) were usually filled with a solution containing the following composition (in mM): KCl 135, BAPTA 5, MgCl_2_ 2, HEPES 10, and Mg-ATP 2 (pH 7.3 adjusted with KOH, osmolarity 290 mOsm; Sigma Aldrich). Voltage-clamp recordings were performed using an Axopatch 200A amplifier (Molecular Devices). Currents were filtered at 2 kHz, digitized (10 kHz) and collected using Clampex 10 (Molecular Devices); the analysis was performed off-line using Clampfit 10 (Molecular Devices). Slicing procedure might activate microglial cells especially near the surface of the slice, whereby recordings were performed on deep cells. Moreover, experiments were performed from 1 to 7 h after slicing. The current/voltage (I/V) relationship of each cell was determined applying voltage steps from -170 to +70 mV (ΔV = 10 mV) for 50 ms holding the cell at -70 mV between steps. Resting membrane potential and membrane capacitance were measured at start of recording. Membrane capacitance was estimated as the total charge (i.e., the current integral, Qstep) mobilized in each cell by a 10 mV depolarizing step (Vstep): Qstep/Vstep (Supplementary Material). Outward and inward rectifier K^+^ current amplitude were evaluated after subtraction of the leak current by a linear fit of the I/V curve between -100 and -50 mV. Cells were considered as expressing the outward rectifier K^+^ current when the I/V relationship showed a rectification above -30 mV and the amplitude measured at 0 mV was at least 10 pA, after leak subtraction; similarly cells showing a small inward rectification below -100 mV were classified as expressing the inward rectifier K^+^ current when subtracted current amplitude was at least 5 pA at -150 mV. Current densities, reported in **Table 1**, were obtained by normalization of current amplitude to cell capacitance. 4-aminopyridine (2 mM, Sigma Aldrich) was used as blocker of outward rectifier K^+^ current.

**Table 1:** Current densities of outward (I_K_; MP = +50 mV) and inward (I_Kir_; MP = -150 mV) rectifier currents of microglia, in hippocampal slices from *Cx3cr1^+/GFP^* and *Cx3cr1^GFP/GFP^* mice at PNW 2 (*p*, *t*-test).

	Cx3cr1^+/GFP^ (n/n_tot_)	Cx3cr1^GFP/GFP^ (n/n_tot_)	*p*
I_K_ (pA/pF)	5.2 ± 0.8 (46/88)	5.6 ± 0.8 (20/56)	0.74
I_Kir_ (pA/pF)	-2.6 ± 0.2 (38/88)	-3.0 ± 0.7 (13/56)	0.5

### Morphological Analysis of Microglial Cells

In slices from perfused brains, endogenous GFP signal was acquired in the hippocampus CA1 stratum radiatum, for a total of 52 serial optical sections (42 μm thickness in the *z*-axis, 129 × 129 μm in xy-axis). Representative images of those fields were obtained as a z-projection based on the maximal intensity signal, using ImageJ software. Within each acquired stack, all the cells that appear entire were subjected to three-dimensional reconstruction.

Three dimensional reconstruction of recorded microglial cells was achieved by injecting biocytin (0.4% dissolved in the internal solution) throughout the recording electrode for at least 10 min. Slices were removed from recording chamber and fixed with paraformaldehyde 4% for 20 min at room temperature and stored at 4°C. After Triton X100 permeabilization, sections were stained with primary antibody at 4°C (anti-GFP antibody, Aves Labs, Inc., Portland, Oregon; 1:800), then incubated with a secondary antibody conjugated to fluorescein (Aves Lab. 1:800), and with streptavidin conjugated to fluorophore Alexa594 (Abcam, 1:800).

Confocal microscopy analysis was performed with a TCS-SP5 (Leica) Laser Scanning System, at 40x magnification. Acquisition files were then processed with ImageJ software for two-dimensional analysis. Three dimensional reconstructions were generated with Imaris software (Bitplane, Zurich, Switzerland) and morphometric analysis of each reconstructed cell, both in acute slices and in perfused brain sections, was performed after surface and volume rendering.

### Isolation of Microglial GFP Positive Cells from Hippocampus

Two weeks old *Cx3cr1^+/GFP^* and *Cx3cr1^GFP/GFP^* mice were anesthetized and decapitated. Brains were removed, and isolated hippocampi were cut into small pieces. Single-cell suspension was achieved by mechanical dissociation and the suspension was applied to a 70 μm cell strainer. Cells were sorted based on GFP expression using a BD FACSAriaIII (BD Biosciences) equipped with a 488 nm laser and FACSDiva software 6.1.3 (BD Biosciences). Briefly, cells were first gated based on forward and side scatter area plot (FSC-A and SSC-A), and then detected in the green fluorescence channel for GFP expression. Following this gate strategy, GFP positive cells sorted were enriched to >98%, used for total RNA isolation with Single Cell RNA Purification Kit (Norgen Biotek Corp., Thorold, ON, Canada), and processed for real-time PCR. The quality and yield of RNAs were verified using the Ultraspec 2000 UV/Visible (Pharmacia Biotech).

### Real-Time PCR

Reverse transcription reaction was performed in a thermocycler (MJ Mini Personal Thermal Cycler; Biorad, Milano, Italy) using IScript TM Reverse Transcription Supermix (Biorad) according to the manufacturer’s protocol, under the following conditions: incubation at 25°C for 5 min, reverse transcription at 42°C for 30 min, inactivation at 85°C for 5 min. Real-time PCR (RT-PCR) was carried out in a I-Cycler IQ Multicolor RT-PCR Detection System (Biorad) using SsoFast EvaGreen Supermix (Biorad) according to the manufacturer’s instructions. The RT-PCR protocol consisted of 40 cycles of denaturation at 95°C for 30 s and annealing/extension at 60°C for 30 s. For quantification analysis the comparative Threshold Cycle (Ct) method was used. The Ct values from each gene were normalized to the Ct value of GAPDH in the same RNA samples. Relative quantification was performed using the 2^-δδCt^ method (Schmittgen and Livak, 2008) and expressed as fold changes in arbitrary values. The following pairs of primers were used: *p2y12:* 5′-CCTGTCGTCAGAGACTACAAG-3′ (F); 5′-GGATTTACTGCGGATCTGAAAG-3′ (R); *p2Y6* 5′-ATCAGCTTCCTGCCTTTCC-3′ (F); 5′-CTGTGAGCCTCTGTAAGAGAGATCG-3′ (R); *cd86:* 5′-AGAACTTACGGAAGCACCCA-3′ (F); 5′-GGCAGATATGCAGTCCCATT-3′ (R); *il-15:* 5′-CATCCATCTCGTGCTACTTGTGTT-3′ (F); 5′-CATCTATCCAGTTGGCCTCTGTTT-3′ (R); *tnf-α :* 5′-GTGGAACTGGCAGAAGAG-3′ (F); 5′-CCATAGAACTGATGAGAGG-3′ (R); *arg-1:* 5′-CTCCAAGCCAAAGTCCTTAGAG-3′ (F); 5′-AGGAGCTGTCATTAGGGACATC-3′ (R); *ym-1* 5′-CAGGTCTGGCAATTCTTCTGAA-3′ (F); 5′-GTCTTGCTCATGTGTGTAAGTGA-3′ (R); *il-1β:* 5′-GCAACTGTTCCTGAACTCAACT-3′ (F); 5′-ATCTTTTGGGGTCCGTCAACT-3′ (R); *cd206:* 5′-CAAGGAAGGTTGGCATTTGT-3′ (F); 5′-CCTTTCAGTCCTTTGCAAGC-3′ (R); *fizz-1:* 5′-CCAATCCAGCTAACTATCCCTCC-3′ (F); 5′-ACCCAGTAGCAGTCATCCCA-3′ (R); *gapdh* 5′-TCGTCCCGTAGACAAAATGG-3′ (F); 5′-TTGAGGTCAATGAAGGGGTC-3′ (R).

### Statistical Analysis

Data, analyzed oﬄine, are presented as mean ± SEM. Statistical analyses were performed using Imaris software for quantitative morphometric analysis; Origin 7 and SigmaPlot 11 (Systat Software Inc., San Jose, CA, USA) software were used for statistical analysis of electrophysiological and imaging data. Paired and unpaired *t*-test and one-way ANOVA were used for parametrical data, as indicated; multiple comparison procedures were performed with Holm-Sidak method. We constructed I/V plots, cumulative distribution plots, and fitted data points by linear or non-linear regression analysis using Origin 7 software. Statistical significance for cumulative distributions was assessed with Kolmogorov-Smirnov test. For statistical analysis of microglial surface areas in fixed brains, we arbitrarily subdivided the cells of each genotype in two classes as smaller or larger respect to the median of each distribution. For statistical analysis of current occurrence in different PNWs and genotypes, statistical difference of proportions was obtained with *z*-test (SigmaPlot 11). For multiple comparisons, multiplicity-adjusted *p* values are indicated on the respective figures when appropriate; otherwise the *p* values were indicated by ^∗^*p* < 0.05, ^∗∗^*p* < 0.01, and ^∗∗∗^*p* < 0.001.

## Results

### Lack of Fractalkine Signaling Impairs Microglial Expression of Voltage-Dependent K^+^ Currents in the Second Postnatal Week

**FIGURE 1:**
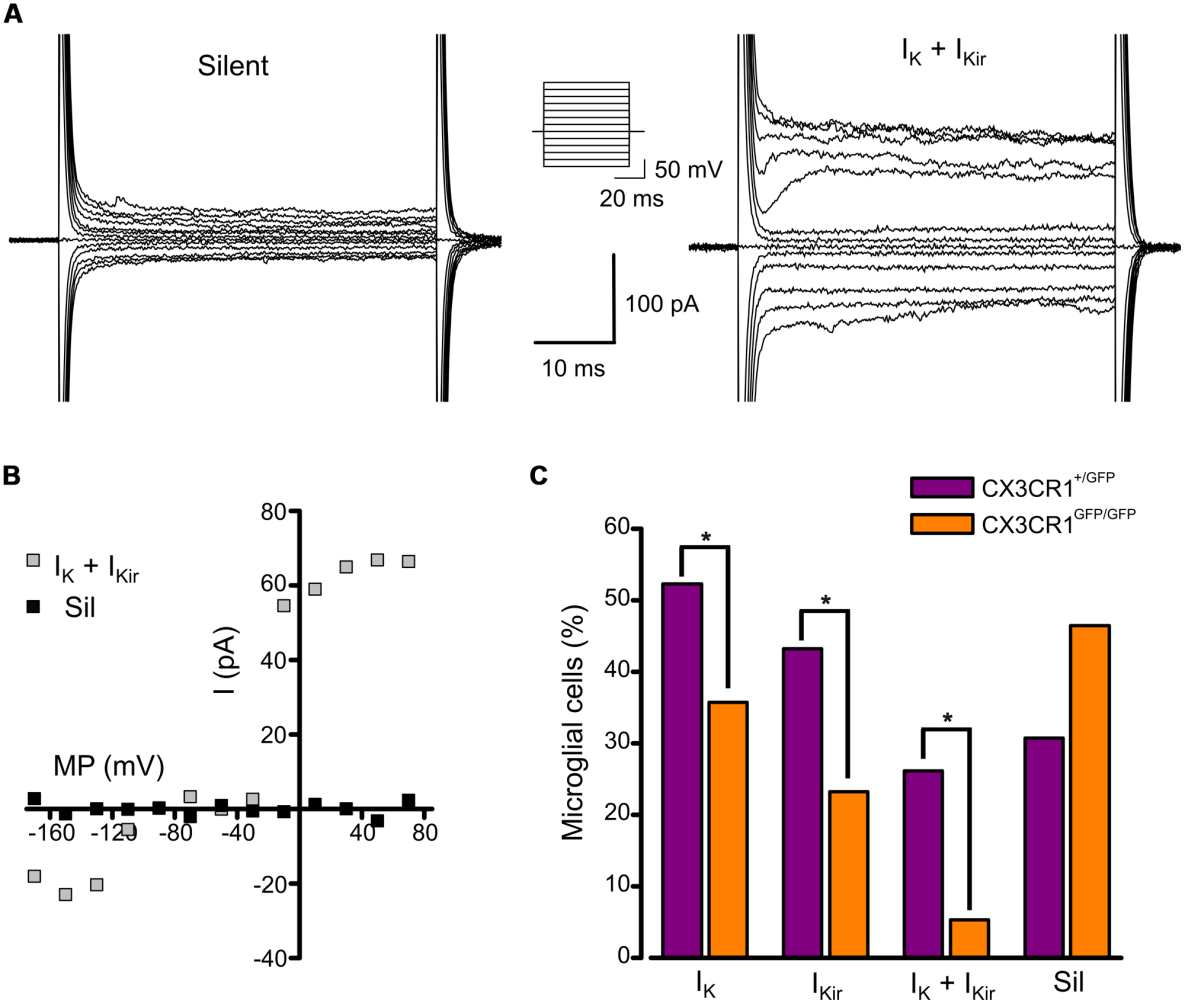
**Expression of voltage-activated K^+^ currents in developing hippocampal microglial from *Cx3cr1^+/GFP^* and *Cx3cr1^GFP/GFP^* mice. (A)** Current traces of representative microglial cells from CX3CR1^+/GFP^ acute slices in response to voltage steps stimulation (steps from -170 to +70 mV, only one out of two steps are shown; holding potential -70 mV). Left: typical current profile of silent microglia. Right: current traces of a microglial cell expressing both I_K_ and I_Kir_. Note that I_K_ expressing cells show a significantly higher membrane capacitance compared to I_K_ negative cells (I_K_ expressing cells 20.0 ± 0.7 pF, *n* = 46; I_K_ negative cells 16.3 ± 0.7 pF, *n* = 42; *p* = 0.001, *t*-test). **(B)** Corresponding current/voltage relationship of the two cells as in A after leak current subtraction. **(C)** Proportion of microglial cells displaying I_K_, I_Kir_, both currents (I_K_ + I_Kir_) or none of them (Sil) in *Cx3cr1*^+/GFP^ (purple bars) and *Cx3cr1*^GFP/GFP^ (orange bars) mice. (^∗^*p* < 0.05, Chi-square test).

We analyzed the electrophysiological properties of microglial cells by whole-cell recordings in CA1 stratum radiatum of acute hippocampal slices from *Cx3cr1^+/GFP^* and *Cx3cr1^GFP/GFP^* mice, during PNW 2. Microglial cells recorded from CX3CR1^+/GFP^ slices, displayed, in different proportion, both outward and inward voltage-dependent K^+^ currents (**Figures 1A,B**). The outward K^+^ current (I_K_, **Figures 1A**, right; 1B), recorded in 46/88 (52%) cells, was blocked by 4-aminopyridine (2 mM; *n* = 3, data not shown). The inward rectifier K^+^ current (I_Kir_, **Figures 1A**, right; 1B) was present in 43% of microglial cells. As shown in **Figure 1C** (purple bars), representing the electrophysiological profile of CX3CR1^+/GFP^ microglia, about one fourth of cells displayed both currents (26%, **Figure 1A**, right), while a similar proportion was silent (30%, **Figure 1A**, left). Microglial cells lacking fractalkine signaling showed a remarkably different electrophysiological profile, with lower frequency of currents expression: I_K_ (36%), I_Kir_ (23%), both currents (5%), silent (46%; **Figure 1C**, orange bars, *p* < 0.05, Chi-square test). However, both I_K_ and I_Kir_ were detected with similar current densities in the two genotypes (**Table 1**).

These results suggest that in the developing hippocampus, a relevant proportion of microglial cells displays a pattern of voltage-dependent K^+^ currents (I_K_ and I_Kir_) resembling that of activated microglia; the occurrence of this phenotype is reduced in mice lacking fractalkine signaling.

### Reduced Ramification of Microglia in *Cx3cr1*^GFP/GFP^ Mice

**FIGURE 2:**
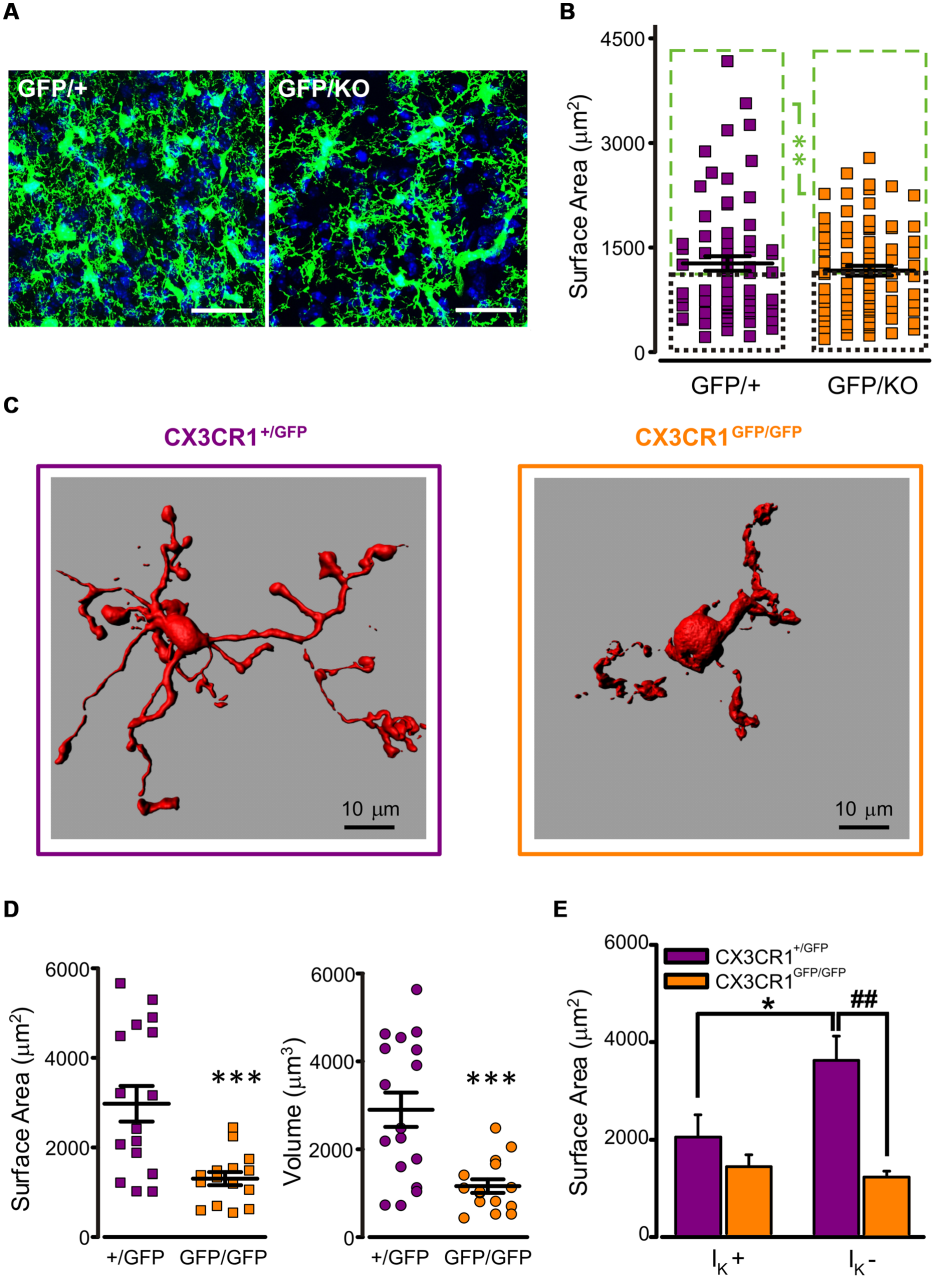
**Reduced ramification of CX3CR1^GFP/GFP^ microglia. (A)** Representative confocal *z*-stack projections showing microglial cells in CA1 stratum radiatum from P8 *Cx3cr1^GFP/+^* (left) and *Cx3cr1^GFP/KO^* (right) mice (blu: DAPI; bar 30 mm). **(B)** Quantitative morphometric analysis of CX3CR1^GFP/+^ and CX3CR1^GFP/KO^ microglial cells; notice that the difference in surface area is limited to large cells (mean surface area in large cells (green boxes): CX3CR1^GFP/+^ 1924 ± 133 μm^2^, *n* = 34; CX3CR1^GFP/KO^ 1705 ± 64 μm^2^, *n* = 43; ^∗∗^ referred to green boxes, *p* < 0.01, two way ANOVA, Holm-Sidak; mean surface area in small cells (black boxes): CX3CR1^GFP/+^ 618 ± 32 μm^2^, *n* = 34; CX3CR1^GFP/KO^ 630 ± 38 μm^2^, *n* = 43, *p* = 0.9, two way ANOVA, Holm-Sidak). **(C)** 3D reconstruction, by Imaris software, of representative biocytin-loaded microglial cells in CA1 stratum radiatum from CX3CR1^+/GFP^ (left) and CX3CR1^GFP/GFP^ (right) slices, showing reduced processes extension in CX3CR1^GFP/GFP^ cells. **(D)** Quantitative morphometric analysis of cell surface area (left) and volume (right) of CX3CR1^+/GFP^ (+/GFP, purple) and CX3CR1^GFP/GFP^ (GFP/GFP, orange) biocytin-loaded microglia. Note that CX3CR1^GFP/GFP^ cells are significantly less extended (mean surface area: CX3CR1^+/GFP^ 2975 ± 398 μm^2^, *n* = 17; CX3CR1^GFP/GFP^ 1302 ± 146 μm^2^, *n* = 15; ^∗∗∗^*p* < 0.0005, *t*-test; mean volume: CX3CR1^+/GFP^ 2899 ± 391 μm^3^, *n* = 17; CX3CR1^GFP/GFP^ 1160 ± 155 μm^3^ *n* = 15; ^∗∗∗^*p* < 0.0005, *t*-test). **(E)** Area correlation between genotypes (CX3CR1^+/GFP^, purple bars; CX3CR1^GFP/GFP^, orange bars) in cells expressing I_K_ (I_K_ +) and cells not expressing I_K_ (I_K_ -). Note that the surface area increase is restricted to CX3CR1^+/GFP^ I_K_ - cells (^∗^*p* < 0.05 vs. I_K_ +; ##*p* < 0.01 vs. CX3CR1^GFP/GFP^, *t*-test).

To characterize microglial morphology and disclose possible differences between the two genotypes, a morphometric analysis was performed in fixed mouse brains of *Cx3cr1^GFP/+^* and *Cx3cr1^GFP/KO^* littermates at P8, both bearing a single GFP copy (**Figure 2A**). The tridimensional reconstruction of microglial cells did not reveal significant differences in cell surface area and volume between the two genotypes (mean surface area: CX3CR1^GFP/+^ 1271 ± 105 μm^2^, *n* = 68; CX3CR1^GFP/KO^ 1168 ± 69 μm^2^, *n* = 86; *p* = 0.4, *t*-test; mean volume: CX3CR1^GFP/+^ 1026 ± 93 μm^3^, *n* = 68; CX3CR1^GFP/KO^ 878 ± 54 μm^3^ *n* = 86; *p* = 0.2, *t*-test). However, a more detailed analysis on the distributions of microglial surface areas showed that a small population of CX3CR1^GFP/+^ cells was characterized by a very large surface area and that this population was absent in CX3CR1^GFP/KO^ brains (**Figure S1**). The subdivision of cells in each genotype in two classes, as smaller (small) or larger (large) respect to the median of each distribution, revealed a significant difference between large cells, with surface area significantly smaller in *Cx3cr1^GFP/KO^* mice (**Figure 2B**; *p* < 0.01, two-way ANOVA, Holm-Sidak) No difference was observed in the small cell group.

Further investigation was carried out on microglial cells from acute hippocampal slices in PNW 2. A subset of microglial cells was filled with biocytin through the recording pipette, allowing high resolution 3D reconstruction and the correlation between electrophysiological profile and morphology. As shown in the representative images in **Figure 2C**, in slices, CX3CR1^GFP/GFP^ cells (right) displayed strikingly reduced processes extension compared to CX3CR1^+/GFP^ cells (left). Quantitative morphometric analysis confirmed that surface area and volume of CX3CR1^GFP/GFP^ microglial cells (*n* = 15) were significantly smaller with respect to those of CX3CR1^+/GFP^ microglia (*n* = 17; *p* < 0.0005, *t*-test; **Figure 2D**).

Moreover, in CX3CR1^+/GFP^ microglial cells, processes extension was negatively correlated with I_K_ expression (**Figure 2E**, purple bars). Indeed, I_K_ positive cells (7/17) showed less ramified morphology and smaller surface area, compared to I_K_ negative cells (*n* = 10/17; *p* < 0.05, *t*-test). In addition, I_K_ positive cells had a significant higher membrane capacitance compared to that of I_K_ negative cells (I_K_ positive cells: 21.7 ± 1.4 pF, *n* = 7; I_K_ negative cells: 16.4 ± 1.4 pF, *n* = 10, *p* < 0.05, *t*-test).

These data point to a subdivision of PNW 2 microglial cells in two functional groups: one resembling *active* microglia, characterized by retracted phenotype and I_K_ expression and another *surveying* microglia, I_K_ negative and ramified. This correlation was not observed in CX3CR1^GFP/GFP^ microglial cells (**Figure 2E**, orange bars). In fact, in brain slices from *Cx3cr1^GFP/GFP^* mice, both I_K_ positive (*n* = 8) and negative (*n* = 7) cells displayed poorly ramified morphology. Consistently, the difference in cell surface area between the two genotypes was restricted to I_K_ negative cells (*p* < 0.01, *t*-test; **Figure 2E**), suggesting that in *Cx3cr1^GFP/GFP^* mice surveying microglia are less ramified and pointing to a functional monitoring defect. None of the two genotypes showed a correlation between I_Kir_ expression and the morphological parameters of surface area and volume (data not shown).

To determine whether the above reported phenotypical differences could rely on cellular polarization, we compared in *Cx3cr1^+/GFP^* and *Cx3cr1^GFP/GFP^* mice the microglial expression profile of specific genes related to M1/M2 activation states. RT-PCR was performed on mRNAs extracted from hippocampal GFP positive cells from the two genotypes. As reported in **Table 2**, no statistically significant differences were observed for either M1 (cd86, il-1β, il-15, tnf-α) or M2 (arg-1, cd206, fizz, ym-1) genes, indicating that the phenotypical changes associated with the lack of fractalkine signaling are not associated with polarization toward “classical” M1 or “alternative” M2 activation states.

**Table 2:** Expression of selected M1/M2 polarization markers, P2y6 and P2y12 in microglial GFP positive cells isolated from hippocampus of *Cx3cr1^+/GFP^* (*n* = 4) and *Cx3cr1^GFP/GFP^* (*n* = 4) mice in the PNW 2.

	CX3CR1^+/GFP^	CX3CR1^GFP/GFP^	*p*
M1 polarization markers			
cd86	1.00 **±**0.32	0.55 **±**0.16	0.23
il-1β	1.00 **±**0.19	1.06 **±**0,16	0.80
il-15	1.00 **±**0.38	1.14 **±**0.29	0.83
tnf-α	1.00 ± 0.22	1.72 ± 0.20	0.06
M2 polarization markers			
arg-1	1.00 ± 0.25	0.63 ± 0.37	0.47
cd206	1.00 ± 0.52	1.03 ± 0.50	0.96
fizz-1	1.00 ± 0.19	0.71 ± 0.12	0.22
ym-1	1.00 ± 0.22	0.65 ± 0.11	0.20
Purinergic receptors			
p2y6	1.00 ± 0.19	1.43 ± 0.29	0.26
p2y12	1.00 ± 0.28	1.47 ± 0.36	0.49

*Transcript levels for CX3CR1^*GFP/GFP*^ microglia are expressed as n-fold increase respect to CX3CR1^+*/GFP*^ (*p*, *t*-test)*.

Altogether, these data indicate that CX3CR1^GFP/GFP^ microglia show functional and morphological differences respect to CX3CR1^+/GFP^ microglia, which are not relying on a different M1/M2 microglial polarization in the two genotypes.

### ATP-Induced Microglial Processes Migration Is Impaired in CX3cr1 Knockout Mice

**FIGURE 3:**
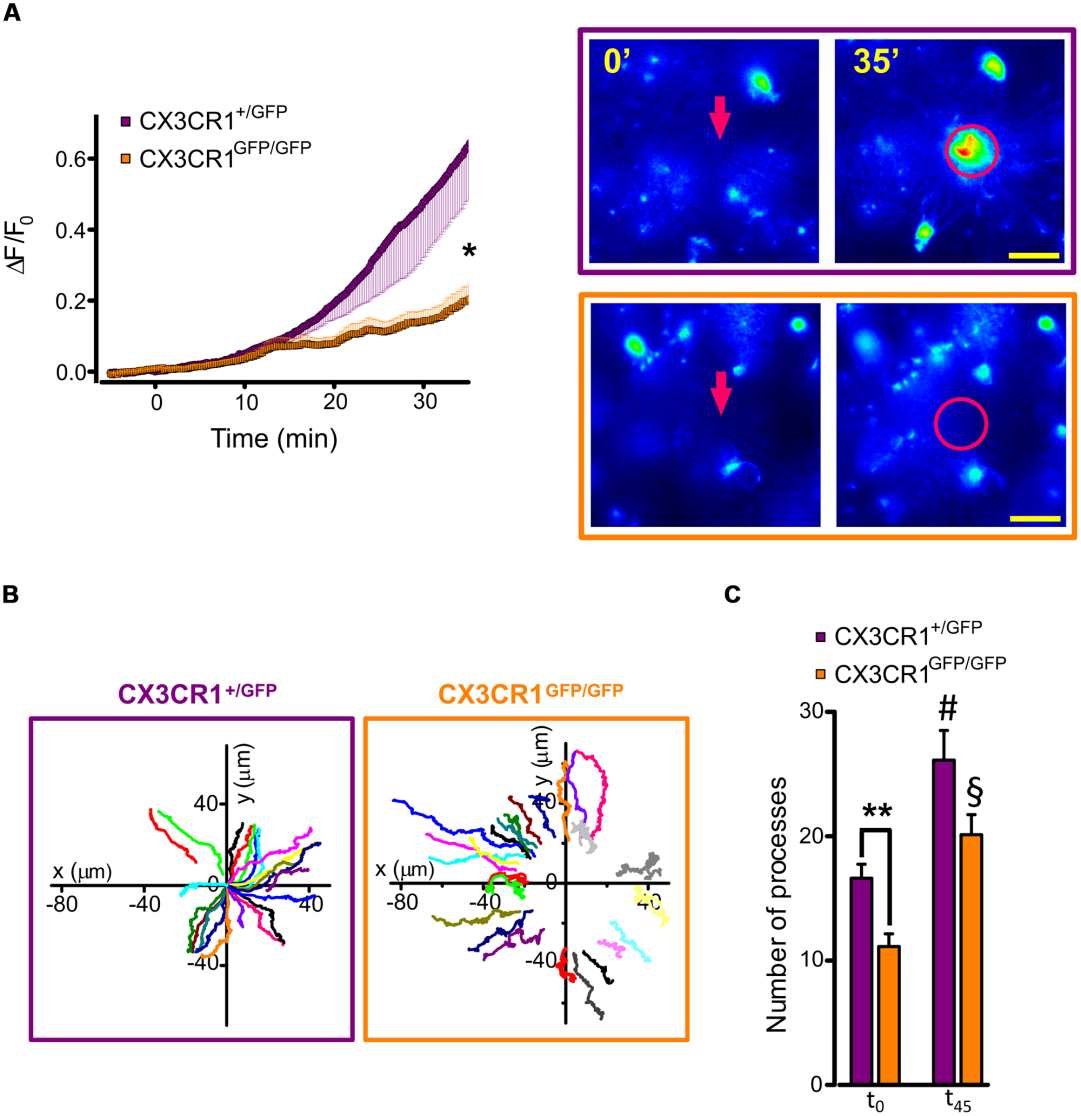
**ATP-induced microglia processes rearrangement in CX3CR1^+/GFP^ and CX3CR1^GFP/GFP^ slices. (A)** Time course (left) of fluorescence ratio (ΔF/F_0_) measured in a circle (10 μm radius) centered on the tip of the ATP puff pipette (arrow), in slices from *Cx3cr1*^+/GFP^ (purple symbols and box, *n* = 18 slices/10 mice) or *Cx3cr1*^GFP/GFP^ mice (orange symbols and box, *n* = 18/5) at PNW 2 (scale bar 20 μm); *t* = 0 corresponds to ATP application (Mg-ATP 3 mM, 5 psi, 100 ms). Note the fluorescence increase in the area around the pipette tip only in CX3CR1+/GFP slices (^∗^*p* < 0.05, unpaired *t*-test). **(B)** Representative tracking of single processes movement in CX3CR1^+/GFP^ (purple box) and CX3CR1^GFP/GFP^ (orange box) fields. Graph origin corresponds to the pipette tip (*x* = 0, *y* = 0). Color traces correspond to single processes. **(C)** Bar chart representing the number of traceable processes in CX3CR1^+/GFP^ (purple bars, *n* = 8 fields) and CX3CR1^GFP/GFP^ (orange bar, *n* = 8 fields) slices at the beginning of the experiments (t_0_) and 45 min after ATP application (t_45_). Note that in CX3CR1^+/GFP^ slices the number of detectable processes at t_0_ is significantly higher than in CX3CR1^GFP/GFP^ slices (^∗∗^*p* < 0.01, *t*-test); after ATP application, processes number increases in both genotypes (#*p* < 0.05 respect to CX3CR1^+/GFP^ at t_0_; §*p* < 0.01 respect to CX3CR1^GFP/GFP^ at t_0_, *t*-test).

Using time-lapse imaging, we characterized the extension of microglial processes toward the focal application of ATP in hippocampal slices. To quantify the directional movement in the two genotypes, we monitored the fluorescence increase in concentric areas around the ATP-containing pipette tip before and after a short ATP puff (3 mM, 100 ms). As shown in **Figure 3A**, the increase in fluorescence measured around the pipette (10 μm radius) was reduced in *Cx3cr1^GFP/GFP^* mice (orange squares, *n* = 18 fields/5 mice) compared to *Cx3cr1^+/GFP^* mice (purple circles, *n* = 18 fields/10 mice; *p* < 0.05, unpaired *t*-test).

To determine whether the impairment in process extension was due to a reduction in expression of ATP receptors, p2y12 and p2y6 mRNAs levels were evaluated by RT-PCR in hippocampal microglial cells. As reported in **Table 2**, no expression differences were observed for either p2y12 or p2y6 transcripts, suggesting that ATP sensing was not altered in developing hippocampal microglial cells lacking *Cx3cr1*.

**FIGURE 4:**
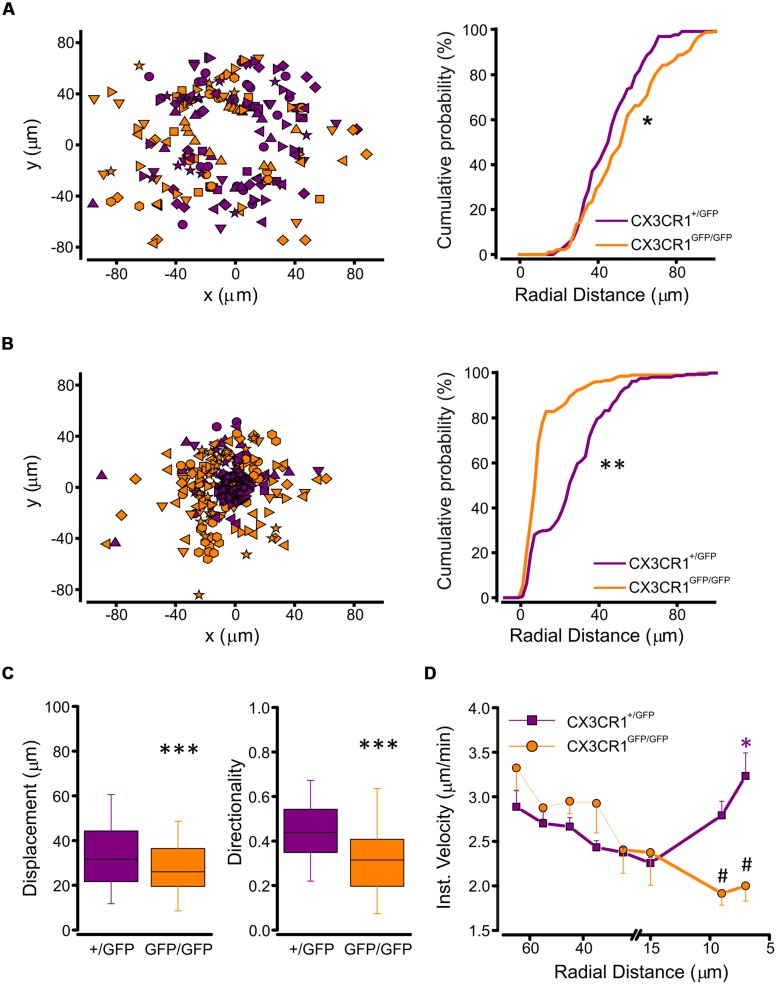
**Tracking analysis of single processes in CX3CR1^+/GFP^ and CX3CR1^GFP/GFP^ microglia. (A)** Left, plot of spatial x-y coordinates respect to the ATP pipette tip of microglial processes in CX3CR1^+/GFP^ (purple symbols, *n* = 133 processes/8 fields) and CX3CR1^GFP/GFP^ hippocampal slices (orange symbols, *n* = 89/8) at start of recordings (t_0_). Right, cumulative distributions of radial distances of microglial processes from the pipette tip in CX3CR1^+/GFP^ (purple) or CX3CR1^GFP/GFP^ (orange) slices, at t_0_. Note that CX3CR1^GFP/GFP^ processes are significantly more distant than CX3CR1^+/GFP^ ones (^∗^*p* < 0.05, Kolmogorov-Smirnov test). **(B)** Left, plot of spatial x-y coordinates of CX3CR1^+/GFP^ (purple symbols, *n* = 209/8) and CX3CR1^GFP/GFP^ (orange symbols, *n* = 161/8) microglial processes 45 min after ATP application (t_45_). Right, cumulative distributions of radial distances of microglial processes in CX3CR1^+/GFP^ (purple) or CX3CR1^GFP/GFP^ (orange) slices, at t_45_. Note that at t_45_ the majority of CX3CR1^GFP/GFP^ processes do not reach the ATP pipette (^∗∗^*p* < 0.0001, Kolmogorov-Smirnov test). **(C)** Box charts of displacement (left) and directionality (right) of CX3CR1^+/GFP^ (purple) and CX3CR1^GFP/GFP^ (orange) microglial processes. In slices from Cx3cr1^+/GFP^ mice displacement and directionality are significantly higher than in those from Cx3cr1^GFP/GFP^ mice (^∗∗∗^*p* < 0.0001, *t*-test), **(D)** Correlation plot showing mean instantaneous velocities vs. radial distance of CX3CR1^+/GFP^ (purple) and CX3CR1^GFP/GFP^ (orange) microglial processes. The velocity of CX3CR1^+/GFP^ processes increase significantly 7 mm far from the ATP pipette tip (^∗^*p* < 0.001, Kruskal-Wallis One Way ANOVA on Ranks, and *p* < 0.05 multiple comparison Dunn’s method versus 15, 25, and 35 μm radial distance). Note that in the proximity of the ATP pipette, the velocity of CX3CR1^GFP/GFP^ processes is significantly slower than that of CX3CR1^+/GFP^ processes (#*p* < 0.01 *t*-test).

To understand the basis of the observed difference in processes extension between the two genotypes, we performed a tracking analysis of single microglial processes (**Figure 3B**; movies 1 and 2 in Supplementary Material). At the start of fluorescence monitoring (t_0_), the number of detected processes was significantly higher in CX3CR1^+/GFP^ than in CX3CR1^GFP/GFP^ slices (*p* < 0.01, *t*-test; **Figure 3C**). Moreover, at t_0_ the process positions in CX3CR1^GFP/GFP^ slices (orange symbols, **Figure 4A**) were more distant from the ATP-containing pipette, compared to CX3CR1^+/GFP^ (purple, *p* < 0.05, Kolmogorov-Smirnov test). The observed differences may reflect the reduced ramification of CX3CR1^GFP/GFP^ microglia, as well as their lower cell density (Paolicelli et al., 2011). Besides, in both genotypes the number of traced processes increased during the experiments, which was significantly higher 45 min after ATP puff (t_45_; **Figure 3C**). We noticed that the mean velocity of processes elongation was similar in the two genotypes (CX3CR1^+/GFP^ 2.39 ± 0.05 μm/min, *n* = 210 processes/8 fields/4 mice; CX3CR1^GFP/GFP^ 2.48 ± 0.08, *n* = 161/8/4; *p* = 0.36; not shown). Nevertheless, the distribution of microglial processes positions was remarkably different between the two genotypes also after ATP-induced extension, as CX3CR1^+/GFP^ processes were significantly closer to the pipette tip at t_45_ (**Figure 4B**; *p* < 0.0001, Kolmogorov-Smirnov test). This was likely due to the lower directionality shown by microglial processes in their elongation. As shown in **Figure 4C**, CX3CR1^GFP/GFP^ processes displayed lower displacement and directionality toward ATP source.

In addition, the correlation between instantaneous velocity and position of single process (measured as radial distance from the ATP source), showed a non-linear profile of extension velocity in CX3CR1^+/GFP^ cells (**Figure 4C**, left). The speed of single process movement increased when processes of CX3CR1^+/GFP^ microglia were in the proximity of the ATP stimulus (**Figure 4D**). This rise in instantaneous velocity as the processes approach the site of ATP application was absent in CX3CR1^GFP/GFP^ slices (**Figure 4D**), contributing to the reduced efficacy of ATP-directed processes rearrangement in the absence of fractalkine signaling.

### Absence of Fractalkine Signaling Affects Developmental Profile of Microglia Functional Properties in the Hippocampus

In order to determine whether the physiological deficits we identified in *Cx3cr1* knockout mice were specific to the early postnatal period, we analyzed the morphological and functional properties of hippocampal microglia at a later developmental stage (PNW 6). Surface area quantification of biocytin-injected microglia, revealed that CX3CR1^+/GFP^ microglia at PNW 6 had significantly greater process extensions relative to PNW 2 (mean surface area = 4662 ± 404 μm^2^, *n* = 15, *p* < 0.01; unpaired *t*-test; **Figure S2**). Conversely, membrane surface area of CX3CR1^GFP/GFP^ microglia at PNW 6 was not greater than at PNW 2 (mean surface area = 1816 ± 286 μm^2^, *n* = 16, *p* = 0.13 PNW 6 vs. PNW 2; unpaired *t*-test) and was significantly smaller when compared to control mice at PNW 6 (*p* < 0.01, *Cx3cr1^GFP/GFP^* vs. *Cx3cr1^+/GFP^* unpaired *t*-test). Moreover, CX3CR1^GFP/GFP^ microglia showed significantly lower capacitance than CX3CR1^+/GFP^ cells (23.7 ± 0.7 pF, *n* = 63; 28.3 ± 0.7 pF, *n* = 63; *p* < 0.001, unpaired *t*-test; PNWs 5–6; **Figure S2**).

**FIGURE 5:**
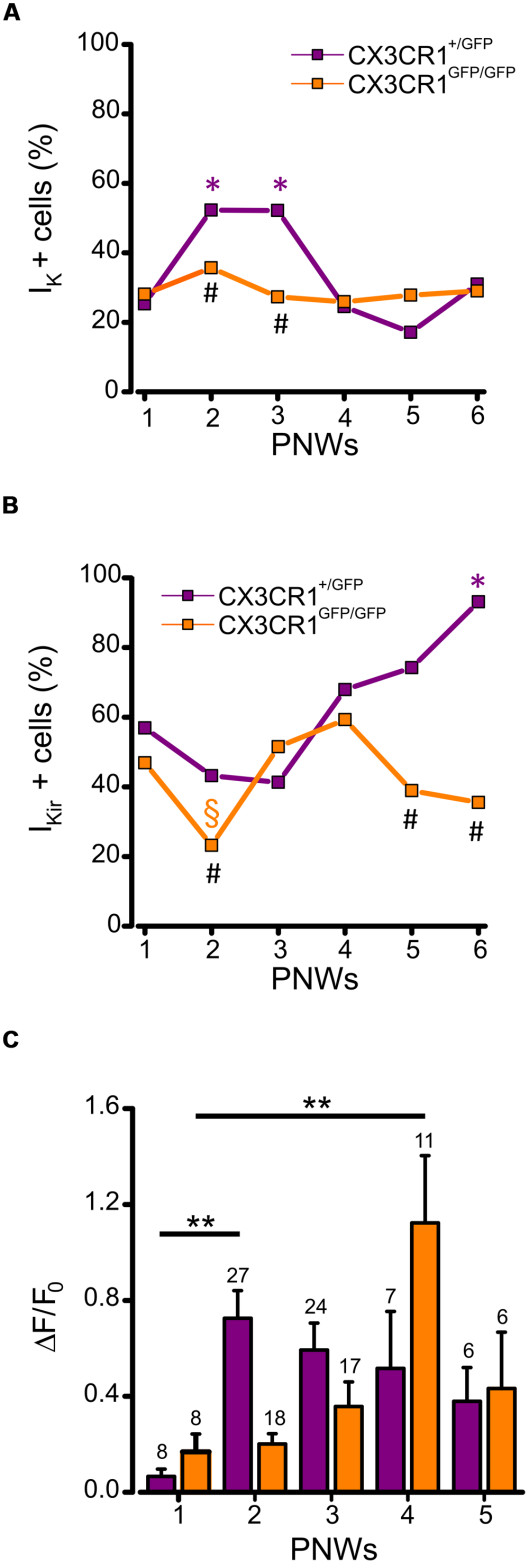
**Developmentally regulated changes in microglial electrophysiological properties and processes rearrangement in CX3CR1^+/GFP^ and CX3CR1^+GFP/GFP^ hippocampal slices. (A)** Time course displaying the occurrence of microglial cells expressing I_K_ (I_K_ + cells) during PNWs 1–6. I_K_ expression transiently increases only in CX3CR^1+/GFP^ microglia (purple symbols) during PNWs 2–3 (*p < 0.05 respect to PNW 1; #p < 0.05 between the two genotypes). (B) Time course displaying the occurrence of microglial cells expressing I_Kir_ (I_Kir_ + cells). I_Kir_ expression transiently decreases only in CX3Cr1^GFP/GFP^ microglia (orange symbols) during PNW 2 (§ *p* < 0.05; * and # as as in panel A). **(C)** Bar chart of mean total fluorescence ratio (ΔF/ΔF_0_) measured during ATP induced processes rearrangement in slices from CX3Cr1^+/GFP^ (purple bars) or CX3Cr1^GFP/GFP^ mice (orange bars) during PNWs 1-5. Number of recorded fields is reported on the top of each bar. Note that the peak of microglial ability to respond to ATP stimulus is delayed in CX3Cr1^GFP/GFP^ mice (**p < 0.01).

We also analyzed the expression of voltage dependent K^+^ currents and ATP-induced process extension during PNWs 1–6. Patch clamp recordings of microglia in CX3CR1^+/GFP^ slices showed that the occurrence of I_K_ was developmentally regulated, transiently increasing in PNWs 2 and 3 (*p* < 0.05 respect to PNW 1, *z*-test; **Figure 5A**, purple). Conversely, in the absence of fractalkine signaling, no developmental increase in I_K_ expression was observed with about one third of cells expressing I_K_ (**Figure 5A**, orange). Consistent with this observation the proportion of cells expressing I_K_ at PNWs 2 and 3 in CX3CR1^+/GFP^ slices was significantly higher than in CX3CR1^GFP/GFP^ slices (*p* < 0.05, **Figure 5A**).

We then tested whether the proportion of I_Kir_ expressing microglia followed a similar temporal pattern. In CX3CR1^+/GFP^ slices, the proportion of microglia displaying I_Kir_ showed a developmental increase to about 90% at PNW 6 (*p* < 0.001 vs. PNWs 1, 2, and 3, *z*-test, **Figure 5B**, purple). Conversely, in CX3CR1^GFP/GFP^ slices this developmental increase of I_Kir_ was not observed, although a transient reduction was found at PNW 2 (**Figure 5B**, orange, *p* < 0.05, vs. PNW 1, *z*-test). Consistently the proportion of I_Kir_ expressing cells in CX3CR1^GFP/GFP^ slices was significantly lower when compared to CX3CR1^+/GFP^ slices at PNWs 5 and 6 (*p* < 0.01, **Figure 5B**). These results show that fractalkine signaling is involved in modulating the profile of microglial voltage-dependent potassium currents during postnatal development.

Finally, we characterized changes in ATP-directed process extension across developmental time points (PNWs 1–5). Quantitative time-lapse imaging analysis revealed that in *Cx3cr1^+/GFP^* mice the speed of extension peaked at PNW 2 (*p* < 0.01, vs PNW 1 ANOVA) with a non-significant tendency to decrease in the following weeks (**Figure 5C**, purple bars). In CX3CR1^GFP/GFP^ slices, a later increase of extension speed was observed, with a peak at PNW 4 (*p* < 0.01, vs. PNW 1 ANOVA; **Figure 5C**, orange bars), pointing to a delay in microglial maturation. These results indicate that fractalkine signaling plays a critical role in the dynamics of maturation of microglia.

## Discussion

We report here that mice lacking *Cx3cr1* are defective in developmentally regulated changes of microglial properties, highlighting the role of fractalkine signaling in neuron-microglia interaction during brain development. Our data show that the lack of CX3CR1 strongly affects both microglial morphology and functional properties. Specifically, microglia in *Cx3cr1^GFP/GFP^* mice were characterized by a less ramified morphology, by the lack of developmental regulation of K^+^ currents and, most remarkably, by defective tissue monitoring, as demonstrated by a reduction in ATP-directed rearrangement.

Our 3D confocal imaging reconstructions from hippocampal slices revealed reduced microglial branching in CX3CR1^GFP/GFP^ slices (**Figure 2**), which was partially confirmed by analysis of perfused brains. CX3CR1 signaling has been shown to influence microglial function under both normal and pathological conditions (Limatola and Ransohoff, 2014; Paolicelli et al., 2014). For example, a transient reduction of microglial density during hippocampal development has been reported in *Cx3cr1* knockout mice (Paolicelli et al., 2011). However, previous studies examining microglia ramification did not evidentiate morphological differences (Liang et al., 2009; Hoshiko et al., 2012), suggesting that they may be the result of the acute hippocampal slices preparation and/or recording conditions, with ATP containing whole cell pipette, used in our studies. The observed difference may, thus, depend on higher susceptibility to insults of *Cx3cr1* knockout mice. Indeed, it is known that several types of brain injury, driving microglia into an activated state, induce stronger response in mice lacking *Cx3cr1* (Cardona et al., 2006). On the other hand, our reconstructions on perfused brains show that morphological differences among the two genotypes might be restricted to a subset of cells showing the largest surface area. It can’t be ruled out that patch clamp recording might have been primarily performed on these cells, as most visible in the slice depth, thus highlighting CX3CR1-dependent morphological defect.

Microglia is characterized by the ability to extend processes in response to ATP, an “alarm signal” released by injured or dying cells (Davalos et al., 2005; Avignone et al., 2008). We found that ATP-directed microglial processes extension was reduced in slices from postnatal hippocampus of *Cx3cr1^GFP/GFP^* mice. This finding is consistent with a previous report showing a reduced ability of microglia to migrate and extend processes in response to focal lesions in *Cx3cr1^GFP/GFP^* mice retina (Liang et al., 2009). Our data point to a defect relying in the lower number and less ramified morphology of microglial cells, rather than in a decrease in the intrinsic motility of processes. Quantitative time-lapse imaging analysis of individual microglial processes showed (i) lower density of processes and (ii) reduced directionality toward ATP in *Cx3cr1^GFP/GFP^* mice, but (iii) similar elongation speed in the two genotypes. Despite stronger GFP expression, CX3CR1^GFP/GFP^ microglial processes are less numerous and are detected at higher distances from the ATP source, compared to CX3CR1^+/GFP^. Moreover, we measured a comparable mean velocity of processes elongation in the two genotypes. Another possible explanation, taking into account the reduced directionality, relies on lower ATP sensitivity of CX3CR1^GFP/GFP^ microglial processes. However, it is unlikely that the observed difference in ATP induced processes extension is due to changes in the expression of purinergic receptors on CX3CR1^GFP/GFP^ microglia (Arnoux et al., 2013), as RT-PCR analysis of hippocampal microglia at PNW 2 showed similar expression of p2y12 and p2y6 transcripts in the two genotypes (**Table 2**).

Furthermore, RT-PCR analysis of the two genotypes did not highlight differences in polarization toward the least (M1) or the most (M2) migratory activation states (Lively and Schlichter, 2013), suggesting that CX3CR1-dependent functions at this stage of development do not involve changes in microglial polarization. It should be considered, however, that our bulk RT-PCR analysis may not have been sufficiently sensitive to detect small variations in gene expression or changes that occurred in a small fraction of cells. Finally, the lack of velocity increase in the close proximity of the ATP pipette tip observed in CX3CR1^GFP/GFP^ processes may depend on longer time needed by knockout processes to reach the ATP-containing pipette. In alternative, it could be a direct effect of CX3CR1 deficiency, as fractalkine signaling is known to promote microglial migration (Maciejewski-Lenoir et al., 1999) and rapidly change in microglia morphology (Liang et al., 2009).

## Conclusion

We can speculate that the observed difference in ATP-induced microglia rearrangement is due to defective branching and directional elongation of microglial processes, pointing to a reduced monitoring capacity of CX3CR1^GFP/GFP^ microglia in the developing hippocampus.

The functional impairment of CX3CR1^GFP/GFP^ microglia is further highlighted by the altered electrophysiological profile observed during postnatal development. Indeed, developing hippocampal microglia display two voltage dependent K^+^ currents: an outward rectifier current, resembling the delayed rectifier K^+^ current described in cultured rat microglia (I_K_) and an inward rectifier K^+^ current (I_Kir_; Nörenberg et al., 1994). Although microglial cells in brain slices are generally considered silent (Boucsein et al., 2000), similar currents have been described in postnatal hippocampus (Schilling and Eder, 2007) and barrel cortex (Arnoux et al., 2013). We made the novel observation that the frequency of occurrence of K^+^ currents in hippocampal microglia changed during postnatal development: I_K_ were more frequent at PNWs 2–3, while I_Kir_ were more frequent at PNW 6. Remarkably, the presence of I_K_ is associated to a retracted morphology and reduced cell surface area resembling that of active microglia, following nerve lesion (Boucsein et al., 2000), epileptic state (Avignone et al., 2008), or LPS treatment (Nörenberg et al., 1994). These data suggest that microglia may have a transient propensity to be activated during hippocampal maturation, as the presence of I_K_ is a typical feature of activated microglia in both slices and dissociated cell cultures, likely associated with the expression of Kv_1.3_ channels (Menteyne et al., 2009; Moussaud et al., 2009). However, RT-PCR analysis does not highlight a clear polarization toward the classical (M1) or alternative (M2) activation states, pointing to a localized phenomenon. In addition, the level of I_K_ functional expression in developing microglia is lower, compared to typically active microglia in pathological contexts (Avignone et al., 2008; Menteyne et al., 2009), suggesting that this regulation may be part of physiological changes, due to varying environmental challenges during development (Eggen et al., 2013). Indeed, developmental regulation of I_K_ has been reported in the barrel cortex (Arnoux et al., 2013). Such phenotypical changes, constituting a sort of developmental activation of microglia (Dalmau et al., 1998), could be induced by local neuronal signals, including cell death or synaptic elimination during developmental circuit refinement (Perry et al., 1985) and participate to the fine tuning of hippocampal synaptic connections (Paolicelli and Gross, 2011).

Strikingly, the occurrence of I_K_ in the hippocampus was not developmentally regulated in mice lacking fractalkine signaling (**Figure 5C**). In this respect, our results differ from a previous report in the barrel cortex of *Cx3cr1^GFP/GFP^* mice, pointing to a delay in microglial maturation, associated with a delay in microglial migration into the barrels (Arnoux et al., 2013). We can speculate that lack of any developmental increase in I_K_ expression in the hippocampus is associated with a loss of environmental influence on microglia maturation in a precise time window.

The expression of I_Kir_ also showed a dynamic developmental profile in control animals (apparently opposite to I_K_ profile), that was absent in *Cx3cr1^GFP/GFP^* mice. This current, typical of dissociated microglia cell cultures (Kettenmann et al., 1990), has been proposed to represent a marker of early microglial activation (Boucsein et al., 2000; Kettenmann et al., 2011). Functional expression of I_Kir_ has been reported in the hippocampus (Schilling and Eder, 2007) and corpus callosum microglia during early postnatal development (Brockhaus et al., 1993), increasing in adult and aging mice (Schilling and Eder, 2014). Under our conditions, I_Kir_ was found in almost all microglia in control animals at PNWs 5 and 6, and was not associated to specific morphological features. Although its function is still unknown, it can be speculated that I_Kir_ frequency is maximal when microglia shows the greatest ramification, thus being maximally capable of sensing tissue signals. The reduced frequency of I_Kir_ in *Cx3cr1^GFP/GFP^* mice could reflect a reduced environmental sensitivity of these cells.

In conclusion, our data support the idea that CX3CR1 deficient microglia is unable to respond properly to specialized environmental signals during normal development (Paolicelli et al., 2014). We speculate that the observed deficit in microglia maturation implies reduced tissue monitoring, suggesting that a developmental microglial defect, partially corrected in the adult, could lead to the delay in synaptic maturation and defective synaptic connectivity reported in these mice (Paolicelli et al., 2011; Hoshiko et al., 2012; Zhan et al., 2014).

## References

[B1] Arnoux I. Hoshiko M. Mandavy L. Avignone E. Yamamoto N. Audinat E. (2013). Adaptive phenotype of microglial cells during the normal postnatal development of the somatosensory “Barrel” cortex. *Glia* 61 1582–1594. 10.1002/glia.22503 23893820

[B2] Avignone E. Ulmann L. Levavasseur F. Rassendren F. Audinat E. (2008). Status epilepticus induces a particular microglial activation state characterized by enhanced purinergic signaling. *J. Neurosci.* 28 9133–9144. 10.1523/JNEUROSCI.1820-08.2008 18784294 PMC6670931

[B3] Bertollini C. Ragozzino D. Gross C. Limatola C. Eusebi F. (2006). Fractalkine/CX3CL1 depresses central synaptic transmission in mouse hippocampal slices. *Neuropharmacology* 51 816–821. 10.1016/j.neuropharm.2006.05.027 16815480

[B4] Biber K. Neumann H. Inoue K. Boddeke H. W. (2007). Neuronal “On” and “Off” signals control microglia. *Trends Neurosci.* 30 596–602. 10.1016/j.tins.2007.08.007 17950926

[B5] Boucsein C. Kettenmann H. Nolte C. (2000). Electrophysiological properties of microglial cells in normal and pathological rat brain slices. *Eur. J. Neurosci.* 12 2049–2058. 10.1046/j.1460-9568.2000.00100.x 10886344

[B6] Brockhaus J. Ilschner S. Banati R. B. Kettenmann H. (1993). Membrane properties of ameboid microglial cells in the corpus callosum slice from early postnatal mice. *J. Neurosci.* 13 4412–4421. 8410196 10.1523/JNEUROSCI.13-10-04412.1993 PMC6576383

[B7] Cardona A. E. Pioro E. P. Sasse M. E. Kostenko V. Cardona S. M. Dijkstra I. M. (2006). Control of microglial neurotoxicity by the fractalkine receptor. *Nat. Neurosci.* 9 917–924. 10.1038/nn1715 16732273

[B8] Crain J. M. Nikodemova M. Watters J. J. (2013). Microglia express distinct M1 and M2 phenotypic markers in the postnatal and adult central nervous system in male and female mice. *J. Neurosci. Res.* 91 1143–1151. 10.1002/jnr.23242 23686747 PMC3715560

[B9] Dalmau I. Finsen B. Zimmer J. González B. Castellano B. (1998). Development of microglia in the postnatal rat hippocampus. *Hippocampus* 8 458–474. 10.1002/(SICI)1098-1063(1998)8:5<458::AID-HIPO6>3.0.CO;2-N 9825958

[B10] Davalos D. Grutzendler J. Yang G. Kim J. V. Zuo Y. Jung S. (2005). ATP mediates rapid microglial response to local brain injury in vivo. *Nat. Neurosci.* 8 752–758. 10.1038/nn1472 15895084

[B11] de Jong E. K. Dijkstra I. M. Hensens M. Brouwer N. van Amerongen M. Liem R. S. (2005). Vesicle-mediated transport and release of CCL21 in endangered neurons: a possible explanation for microglia activation remote from a primary lesion. *J. Neurosci.* 25 7548–7557. 10.1523/JNEUROSCI.1019-05.2005 16107642 PMC6725403

[B12] Eder C. (2005). Regulation of microglial behavior by ion channel activity. *J. Neurosci. Res.* 81 314–321. 10.1002/jnr.20476 15929071

[B13] Eder C. Klee R. Heinemann U. (1998). Involvement of stretch-activated Cl-channels in ramification of murine microglia. *J. Neurosci.* 18 7127–7137. 9736636 10.1523/JNEUROSCI.18-18-07127.1998 PMC6793253

[B14] Eggen B. J. Raj D. Hanisch U. K. Boddeke H. W. (2013). Microglial phenotype and adaptation. *J. Neuroimmune Pharmacol.* 8 807–823. 10.1007/s11481-013-9490-4 23881706

[B15] Harrison J. K. Jiang Y. Chen S. Xia Y. Maciejewski D. McNamara R. K. (1998). Role for neuronally derived fractalkine in mediating interactions between neurons and CX3CR1-expressing microglia. *Proc. Natl. Acad. Sci. U.S.A.* 95 10896–10901. 10.1073/pnas.95.18.10896 9724801 PMC27992

[B16] Haskell C. A. Hancock W. W. Salant D. J. Gao W. Csizmadia V. Peters W. (2001). Targeted deletion of CX(3)CR1 reveals a role for fractalkine in cardiac allograft rejection. *J. Clin. Invest.* 108 679–688. 10.1172/JCI12976 11544273 PMC209384

[B17] Hoshiko M. Arnoux I. Avignone E. Yamamoto N. Audinat E. (2012). Deficiency of the microglial receptor CX3CR1 impairs postnatal functional development of thalamocortical synapses in the barrel cortex. *J. Neurosci.* 32 15106–15111. 10.1523/JNEUROSCI.1167-12.2012 23100431 PMC6704837

[B18] Inoue K. Koizumi S. Tsuda M. (2007). The role of nucleotides in the neuron–glia communication responsible for the brain functions. *J. Neurochem.* 102 1447–1458. 10.1111/j.1471-4159.2007.04824.x 17697046

[B19] Jung S. Aliberti J. Graemmel P. Sunshine M. J. Kreutzberg G. W. Sher A. (2000). Analysis of fractalkine receptor CX(3)CR1 function by targeted deletion and green fluorescent protein reporter gene insertion. *Mol. Cell. Biol.* 20 4106–4114. 10.1128/MCB.20.11.4106-4114.2000 10805752 PMC85780

[B20] Kettenmann H. Hanisch U. K. Noda M. Verkhratsky A. (2011). Physiology of microglia. *Physiol. Rev.* 91 461–553. 10.1152/physrev.00011.2010 21527731

[B21] Kettenmann H. Hoppe D. Gottmann K. Banati R. Kreutzberg G. (1990). Cultured microglial cells have a distinct pattern of membrane channels different from peritoneal macrophages. *J. Neurosci. Res.* 26 278–287. 10.1002/jnr.490260303 1697905

[B22] Kotecha S. A. Schlichter L. C. (1999). A Kv1.5 to Kv1.3 switch in endogenous hippocampal microglia and a role in proliferation. *J. Neurosci*. 19 10680–10693. 10594052 10.1523/JNEUROSCI.19-24-10680.1999 PMC6784954

[B23] Lauro C. Di Angelantonio S. Cipriani R. Sobrero F. Antonilli L. Brusadin V. (2008). Activity of adenosine receptors type 1 is required for CX3CL1-mediated neuroprotection and neuromodulation in hippocampal neurons. *J. Immunol.* 180 7590–7596. 10.4049/jimmunol.180.11.7590 18490761

[B24] Lee J. E. Liang K. J. Fariss R. N. Wong W. T. (2008). Ex vivo dynamic imaging of retinal microglia using time-lapse confocal microscopy. *Invest. Ophthalmol. Vis. Sci.* 49 4169–4176. 10.1167/iovs.08-2076 18487378 PMC2652634

[B25] Liang K. J. Lee J. E. Wang Y. D. Ma W. Fontainhas A. M. Fariss R. N. (2009). Regulation of dynamic behavior of retinal microglia by CX3CR1 signaling. *Invest. Ophthalmol. Vis. Sci.* 50 4444–4451. 10.1167/iovs.08-3357 19443728 PMC2749316

[B26] Limatola C. Lauro C. Catalano M. Ciotti M. T. Bertollini C. Di Angelantonio S. (2005). Chemokine CX3CL1 protects rat hippocampal neurons against glutamate-mediated excitotoxicity. *J. Neuroimmunol.* 166 19–28. 10.1016/j.jneuroim.2005.03.023 16019082

[B27] Limatola C. Ransohoff R. M. (2014). Modulating neurotoxicity through CX3CL1/CX3CR1 signaling. *Front. Cell. Neurosci.* 8:229 10.3389/fncel.2014.00229 PMC4126442 25152714

[B28] Lively S. Schlichter L. C. (2013). The microglial activation state regulates migration and roles of matrix-dissolving enzymes for invasion. *J. Neuroinflammation.* 10:75 10.1186/1742-2094-10-75 PMC3693964 23786632

[B29] Maciejewski-Lenoir D. Chen S. Feng L. Maki R. Bacon K. B. (1999). Characterization of fractalkine in rat brain cells: migratory and activation signals for CX3CR-1-expressing microglia. *J. Immunol.* 163 1628–1635. 10415068

[B30] Maggi L. Scianni M. Branchi I. D’Andrea I. Lauro C. Limatola C. (2011). CX(3)CR1 deficiency alters hippocampal-dependent plasticity phenomena blunting the effects of enriched environment. *Front. Cell. Neurosci.* 5:22 10.3389/fncel.2011.00022 PMC3198035 22025910

[B31] Menteyne A. Levavasseur F. Audinat E. Avignone E. (2009). Predominant functional expression of Kv1.3 by activated microglia of the hippocampus after status epilepticus. *PLoS ONE* 4:e6770 10.1371/journal.pone.0006770 PMC2727945 19707551

[B32] Mizutani M. Pino P. A. Saederup N. Charo I. F. Ransohoff R. M. Cardona A. E. (2012). The fractalkine receptor but not CCR2 is present on microglia from embryonic development throughout adulthood. *J. Immunol.* 188 29–36. 10.4049/jimmunol.1100421 22079990 PMC3244524

[B33] Moussaud S. Lamodière E. Savage C. Draheim H. J. (2009). Characterisation of K^+^ currents in the C8-B4 microglial cell line and their regulation by microglia activating stimuli. *Cell. Physiol. Biochem.* 24 141–152. 10.1159/000233240 19710528

[B34] Nimmerjahn A. Kirchhoff F. Helmchen F. (2005). Resting microglial cells are highly dynamic surveillants of brain parenchyma in vivo. *Science* 308 1314–1318. 10.1126/science.1110647 15831717

[B35] Nörenberg W. Gebicke-Haerter P. J. Illes P. (1994). Voltage-dependent potassium channels in activated rat microglia. *J. Physiol.* 475 15–32. 10.1113/jphysiol.1994.sp020046 7514664 PMC1160352

[B36] Olah M. Amor S. Brouwer N. Vinet J. Eggen B. Biber K. (2012). Identification of a microglia phenotype supportive of remyelination. *Glia* 60 306–321. 10.1002/glia.21266 22072381

[B37] Paolicelli R. C. Bisht K. Tremblay M. E. (2014). Fractalkine regulation of microglial physiology and consequences on the brain and behavior. *Front. Cell. Neurosci.* 8:129 10.3389/fncel.2014.00129 PMC4026677 24860431

[B38] Paolicelli R. C. Bolasco G. Pagani F. Maggi L. Scianni M. Panzanelli P. (2011). Synaptic pruning by microglia is necessary for normal brain development. *Science* 333 1456–1458. 10.1126/science.1202529 21778362

[B39] Paolicelli R. C. Gross C. T. (2011). Microglia in development: linking brain wiring to brain environment. *Neuron Glia Biol*. 7 77–83. 10.1017/S1740925X12000105 22857738

[B40] Perry V. H. Hume D. A. Gordon S. (1985). Immunohistochemical localization of macrophages and microglia in the adult and developing mouse brain. *Neuroscience* 15 313–326. 10.1016/0306-4522(85)90215-5 3895031

[B41] Piccinin S. Di Angelantonio S. Piccioni A. Volpini R. Cristalli G. Fredholm B. B. (2010). CX3CL1-induced modulation at CA1 synapses reveals multiple mechanisms of EPSC modulation involving adenosine receptor subtypes. *J. Neuroimmunol.* 224 85–92. 10.1016/j.jneuroim.2010.05.012 20570369

[B42] Ponomarev E. D. Maresz K. Tan Y. Dittel B. N. (2007). CNS-derived interleukin-4 is essential for the regulation of autoimmune inflammation and induces a state of alternative activation in microglial cells. *J. Neurosci.* 27 10714–10721. 10.1523/JNEUROSCI.1922-07.2007 17913905 PMC6672829

[B43] Prinz M. Mildner A. (2011). Microglia in the CNS: immigrants from another world. *Glia* 59 177–187. 10.1002/glia.21104 21125659

[B44] Ragozzino D. Di Angelantonio S. Trettel F. Bertollini C. Maggi L. Gross C. (2006). Chemokine fractalkine/CX3CL1 negatively modulates active glutamatergic synapses in rat hippocampal neurons. *J. Neurosci.* 26 10488–10498. 10.1523/JNEUROSCI.3192-06.2006 17035533 PMC6674698

[B45] Raivich G. (2005). Like cops on the beat: the active role of resting microglia. *Trends Neurosci*. 28 571–573. 10.1016/j.tins.2005.09.001 16165228

[B46] Rappert A. Biber K. Nolte C. Lipp M. Schubel A. Lu B. (2002). Secondary lymphoid tissue chemokine (CCL21) activates CXCR3 to trigger a Cl-current and chemotaxis in murine microglia. *J. Immunol.* 168 3221–3226. 10.4049/jimmunol.168.7.3221 11907075

[B47] Rogers J. T. Morganti J. M. Bachstetter A. D. Hudson C. E. Peters M. M. Grimmig B. A. (2011). CX3CR1 deficiency leads to impairment of hippocampal cognitive function and synaptic plasticity. *J. Neurosci.* 31 16241–16250. 10.1523/JNEUROSCI.3667-11.2011 22072675 PMC3236509

[B48] Schafer D. P. Lehrman E. K. Kautzman A. G. Koyama R. Mardinly A. R. Yamasaki R. (2012). Microglia sculpt postnatal neural circuits in an activity and complement-dependent manner. *Neuron* 74 691–705. 10.1016/j.neuron.2012.03.026 22632727 PMC3528177

[B49] Schilling T. Eder C. (2007). Ion channel expression in resting and activated microglia of hippocampal slices from juvenile mice. *Brain Res.* 1186 21–28. 10.1016/j.brainres.2007.10.027 18005942

[B50] Schilling T. Eder C. (2014). Microglial K^+^ channel expression in young adult and aged mice. *Glia* 63 664–672. 10.1002/glia.22776 25472417 PMC4359010

[B51] Schilling T. Stock C. Schwab A. Eder C. (2004). Functional importance of Ca2^+^-activated K^+^ channels for lysophosphatidic acid-induced microglial migration. *Eur. J. Neurosci.* 19 1469–1474. 10.1111/j.1460-9568.2004.03265.x 15066143

[B52] Schindelin J. Arganda-Carreras I. Frise E. Kaynig V. Longair M. Pietzsch T. (2012). Fiji: an open-source platform for biological-image analysis. *Nat. Methods* 9 676–682. 10.1038/nmeth.2019 22743772 PMC3855844

[B53] Schlichter L. C. Sakellaropoulos G. Ballyk B. Pennefather P. S. Phipps D. J. (1996). Properties of K^+^ and Cl–channels and their involvement in proliferation of rat microglial cells. *Glia* 17 225–236. 10.1002/(SICI)1098-1136(199607)17:3<225::AID-GLIA5>3.0.CO;2-# 8840164

[B54] Schmittgen T. D. Livak K. J. (2008). Analyzing real-time PCR data by the comparative C(T)method. *Nat. Protoc*. 3 1101–1108. 10.1038/nprot.2008.73 18546601

[B55] Schneider C. A. Rasband W. S. Eliceiri K. W. (2012). NIH Image to ImageJ: 25 years of image analysis. *Nat. Methods* 9 671–675. 10.1038/nmeth.2089 22930834 PMC5554542

[B56] Tarozzo G. Bortolazzi S. Crochemore C. Chen S. C. Lira A. S. Abrams J. S. (2003). Fractalkine protein localization and gene expression in mouse brain. *J. Neurosci. Res.* 73 81–88. 10.1002/jnr.10645 12815711

[B57] Tremblay M. È. Lowery R. L. Majewska A. K. (2010). Microglial interactions with synapses are modulated by visual experience. *PLoS Biol.* 8:e1000527 10.1371/journal.pbio.1000527 PMC2970556 21072242

[B58] Wake H. Moorhouse A. J. Jinno S. Kohsaka S. Nabekura J. (2009). Resting microglia directly monitor the functional state of synapses in vivo and determine the fate of ischemic terminals. *J. Neurosci.* 29 3974–3980. 10.1523/JNEUROSCI.4363-08.2009 19339593 PMC6665392

[B59] Walker F. R. Beynon S. B. Jones K. A. Zhao Z. Kongsui R. Cairns M. (2014). Dynamic structural remodelling of microglia in health, and disease: a review of the models, the signals, and the mechanisms. *Brain Behav. Immun*. 37 1–14. 10.1016/j.bbi.2013.12.010 24412599

[B60] Wu C. Asokan S. B. Berginski M. E. Haynes E. M. Sharpless N. E. Griffith J. D. (2012). Arp2/3 is critical for lamellipodia and response to extracellular matrix cues but is dispensable for chemotaxis. *Cell* 148 973–987. 10.1016/j.cell.2011.12.034 22385962 PMC3707508

[B61] Zhan Y. Paolicelli R. C. Sforazzini F. Weinhard L. Bolasco G. Pagani F. (2014). Deficient neuron-microglia signaling results in impaired functional brain connectivity and social behavior. *Nat. Neurosci*. 17 400–406. 10.1038/nn.3641 24487234

