# Supplementary material for: Defective microglial development in the hippocampus of Cx3cr1 deficient mice
Source: Front Cell Neurosci. 2015 Mar 31;9:111. doi: 10.3389/fncel.2015.00111 (PMC4379915; doi:10.3389/fncel.2015.00111)
Supplement: Supplementary file 3 [file presentation_1.pptx]

## Slide 1
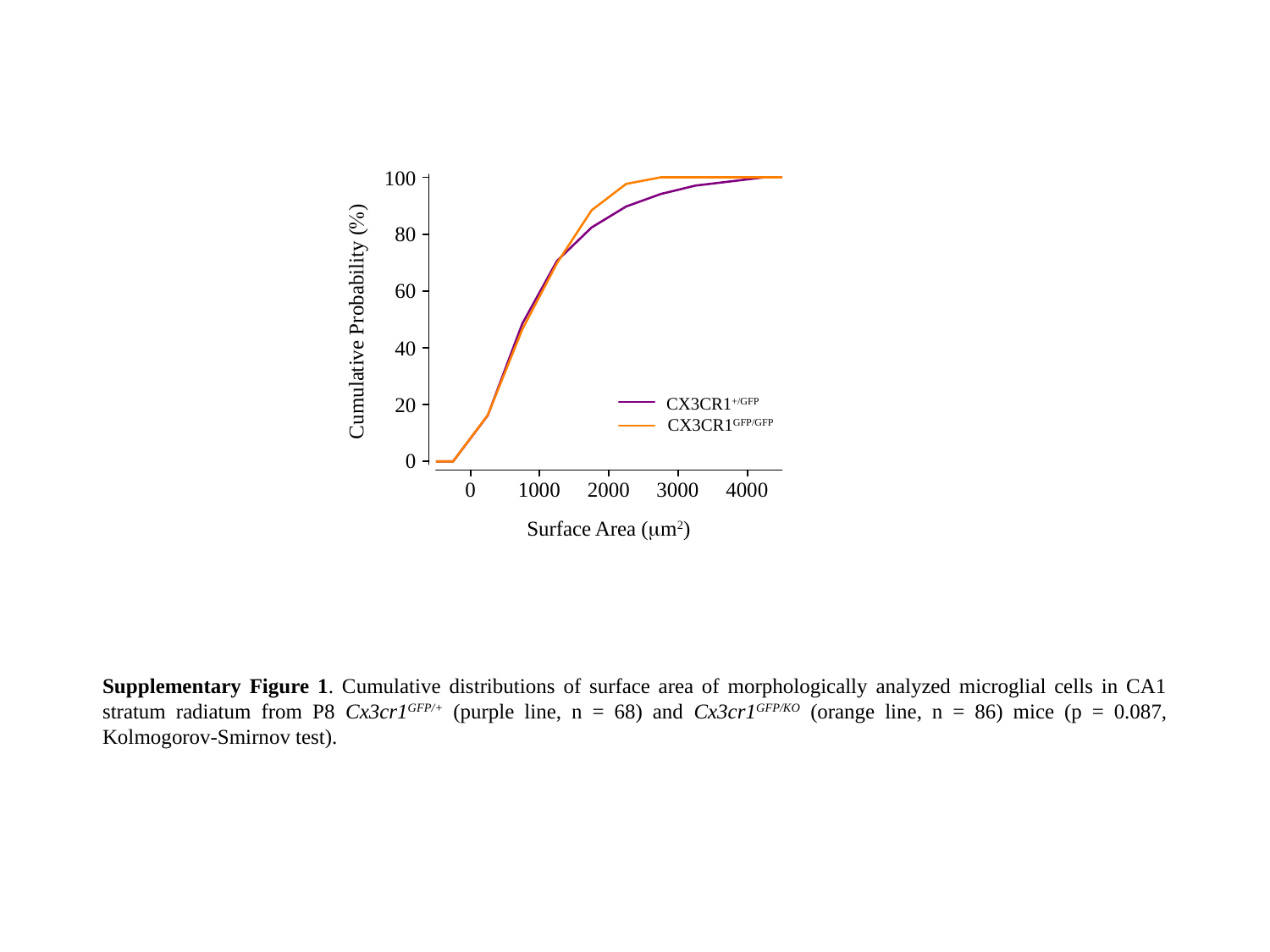

100
80
60
Cumulative Probability (%)
40
20
CX3CR1+/GFP
CX3CR1GFP/GFP
0
0
1000
2000
3000
4000
Surface Area (mm2)
Supplementary Figure 1. Cumulative distributions of surface area of morphologically analyzed microglial cells in CA1 stratum radiatum from P8 Cx3cr1GFP/+ (purple line, n = 68) and Cx3cr1GFP/KO (orange line, n = 86) mice (p = 0.087, Kolmogorov-Smirnov test).
