# Supplementary material for: Defective microglial development in the hippocampus of Cx3cr1 deficient mice
Source: Front Cell Neurosci. 2015 Mar 31;9:111. doi: 10.3389/fncel.2015.00111 (PMC4379915; doi:10.3389/fncel.2015.00111)
Supplement: Supplementary file 4 [file presentation_2.pptx]

## Slide 1
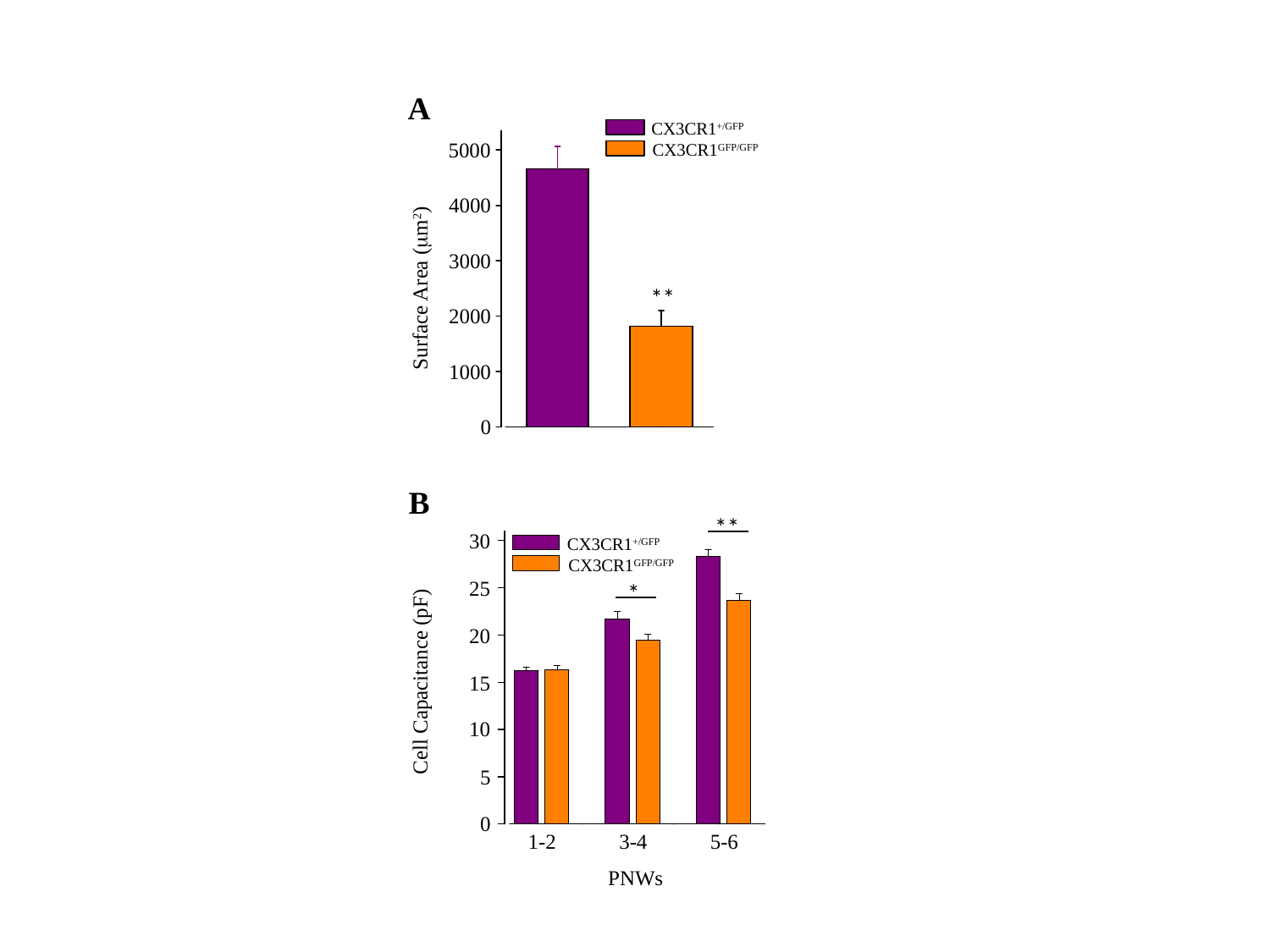

A
CX3CR1+/GFP
5000
4000
3000
2000
1000
0
CX3CR1GFP/GFP
Surface Area (mm2)
**
B
**
30
25
20
15
10
5
0
CX3CR1+/GFP
CX3CR1GFP/GFP
*
Cell Capacitance (pF)
1-2
3-4
5-6
PNWs

## Slide 2
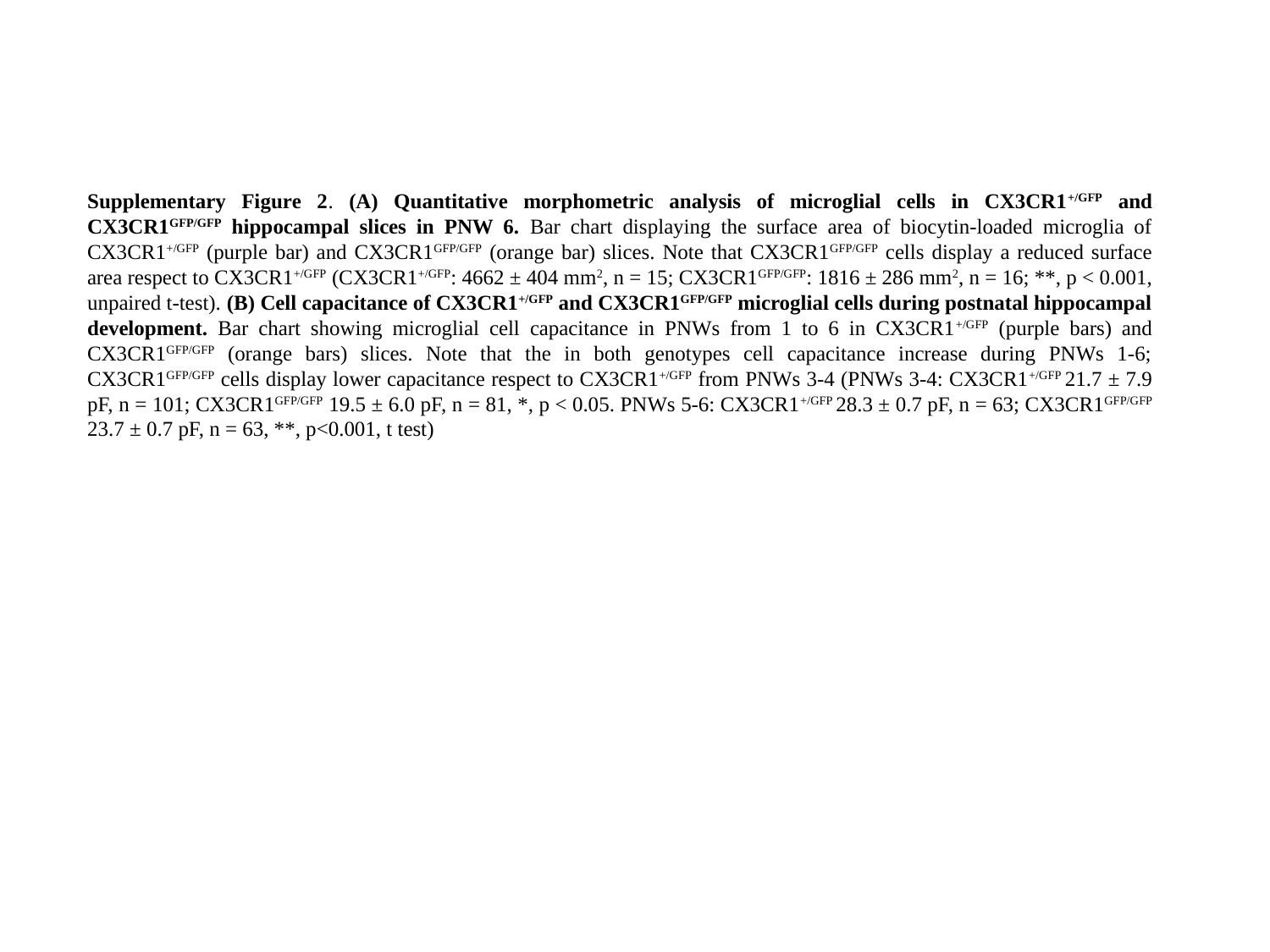

Supplementary Figure 2. (A) Quantitative morphometric analysis of microglial cells in CX3CR1+/GFP and CX3CR1GFP/GFP hippocampal slices in PNW 6. Bar chart displaying the surface area of biocytin-loaded microglia of CX3CR1+/GFP (purple bar) and CX3CR1GFP/GFP (orange bar) slices. Note that CX3CR1GFP/GFP cells display a reduced surface area respect to CX3CR1+/GFP (CX3CR1+/GFP: 4662 ± 404 mm2, n = 15; CX3CR1GFP/GFP: 1816 ± 286 mm2, n = 16; **, p < 0.001, unpaired t-test). (B) Cell capacitance of CX3CR1+/GFP and CX3CR1GFP/GFP microglial cells during postnatal hippocampal development. Bar chart showing microglial cell capacitance in PNWs from 1 to 6 in CX3CR1+/GFP (purple bars) and CX3CR1GFP/GFP (orange bars) slices. Note that the in both genotypes cell capacitance increase during PNWs 1-6; CX3CR1GFP/GFP cells display lower capacitance respect to CX3CR1+/GFP from PNWs 3-4 (PNWs 3-4: CX3CR1+/GFP 21.7 ± 7.9 pF, n = 101; CX3CR1GFP/GFP 19.5 ± 6.0 pF, n = 81, *, p < 0.05. PNWs 5-6: CX3CR1+/GFP 28.3 ± 0.7 pF, n = 63; CX3CR1GFP/GFP 23.7 ± 0.7 pF, n = 63, **, p<0.001, t test)
